# 2,5-Dioxopyrrolidin-1-yl 3-(furan-2-yl)acrylate

**DOI:** 10.1107/S1600536811040566

**Published:** 2011-10-08

**Authors:** Haifeng Zhu, Miao Guo, Han Chen, Haixiao Jin, Hongze Liang

**Affiliations:** a Faculty of Materials Science and Chemical Engineering, Ningbo University, Ningbo, Zhejiang 315211, People’s Republic of China

## Abstract

The title compound, C_11_H_9_NO_5_, was prepared by the reaction of 2-furan­acrylic acid and *N*-hy­droxy­succinimide. The mol­ecule consists of two approximately planar moieties, *viz.* a succinimide group and the rest of the mol­ecule [the largest deviations from the least-squares planes are 0.120 (1) and 0.210 (1) Å, respectively]. The dihedral angle between these fragments is 63.70 (5)°. In the crystal, mol­ecules are linked by C—H⋯O hydrogen bonds into two-dimensional nets.

## Related literature

For derivatives of *N*-hy­droxy­succinimide, see: Anderson *et al.* (1964 ▶); Blumberg & Vallee (1975 ▶); Brown *et al.* (2005 ▶); Cheng *et al.* (2007 ▶); Jones (2003 ▶).
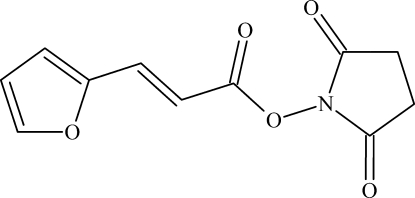


## Experimental

### 

#### Crystal data

C_11_H_9_NO_5_
*M* *_r_* = 235.19
Orthorhombic, 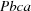

*a* = 10.3054 (13) Å
*b* = 9.2376 (12) Å
*c* = 21.892 (3) Å
*V* = 2084.0 (5) Å^3^
*Z* = 8
Mo *K*α radiation
μ = 0.12 mm^−1^
*T* = 296 K
0.30 × 0.30 × 0.10 mm

#### Data collection

Bruker APEXII CCD area-detector diffractometer
Absorption correction: multi-scan (*SADABS*; Bruker, 2005 ▶) *T*_min_ = 0.977, *T*_max_ = 0.977
16939 measured reflections
2399 independent reflections
1900 reflections with *I* > 2σ(*I*)
*R*_int_ = 0.041

#### Refinement

*R*[*F* ^2^ > 2σ(*F* ^2^)] = 0.035
*wR*(*F* ^2^) = 0.082
*S* = 1.02
2399 reflections
154 parameters
H-atom parameters constrained
Δρ_max_ = 0.20 e Å^−3^
Δρ_min_ = −0.24 e Å^−3^

### 

Data collection: *APEX2* (Bruker, 2005 ▶); cell refinement: *SAINT* (Bruker, 2005 ▶); data reduction: *SAINT*; program(s) used to solve structure: *SHELXS97* (Sheldrick, 2008 ▶); program(s) used to refine structure: *SHELXL97* (Sheldrick, 2008 ▶); molecular graphics: *SHELXTL* (Sheldrick, 2008 ▶); software used to prepare material for publication: *SHELXTL*.

## Supplementary Material

Crystal structure: contains datablock(s) I, global. DOI: 10.1107/S1600536811040566/yk2020sup1.cif

Structure factors: contains datablock(s) I. DOI: 10.1107/S1600536811040566/yk2020Isup2.hkl

Supplementary material file. DOI: 10.1107/S1600536811040566/yk2020Isup3.cml

Additional supplementary materials:  crystallographic information; 3D view; checkCIF report

## Figures and Tables

**Table 1:** Hydrogen-bond geometry (Å, °)

*D*—H⋯*A*	*D*—H	H⋯*A*	*D*⋯*A*	*D*—H⋯*A*
C12—H12*A*⋯O1^i^	0.93	2.48	3.3533 (17)	156
C14—H14*A*⋯O5^ii^	0.93	2.45	3.3672 (18)	169

Symmetry codes: (i) 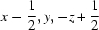; (ii) 
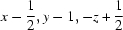
.

**Fig. 1.**
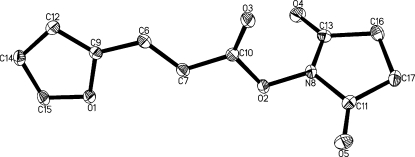
The molecular structure of (I) showing displacement ellipsoids at the 30% probability level.

## supplementary crystallographic information

### Comment

*N*-Hydroxysuccinimide is frequently used in organic chemistry as an activating reagent, which readily form crystalline adducts with amines or acids (Jones, 2003). *N*-Hydroxysuccinimide esters are widely used as leaving groups to activate carboxylic acids (Cheng *et al.*, 2007). The title compound *N*-(2-furanacryloyl)-succinimide ester is an intermediate of FAPGG which is the substrate of diagnostic reagent. We have used a simple procedure to synthesize the title compound in reasonable yields (Blumberg & Vallee, 1975; Brown *et al.*, 2005).

The molecular structure of the title compound (I) is shown in Fig.1. In the molecule, the dihedral angle between the furan and succinimide rings is 56.26 (43)°. In the crystal structure, molecules are linked by the C12—H12···O1^i^ hydrogen bonds to form chains along *a* and C14—H14···O5^ii^ hydrogen bonds to form chains along *b* directions.

### Experimental

2-Furanacrylic acid (13.81 g, 0.10 mol), *N*-hydroxysuccinimide (11.51 g, 0.10 mol) and dicyclohexylcarbodiimide (20.63 g, 0.10 mol) were added to 200 ml dioxane in a round flask. This mixture was stirred at 4°C for 14 h before the dicyclohexylurea was removed by filtration. Then the resulting dark brown filtrate was evaporated in vacuum to give the dark brown residue. Slight brown crystals were obtained by recrystallization from 2-propanol (15.29 g, 65%). 1H NMR(400 MHz, CDCl3): \d 7.62 (d, J=16 Hz, 1H), 7.56 (d, J=1.6 Hz, 1H), 6.77 (d, J=3.6 Hz, 1H), 6.53–6.52 (dd, J=3.6 Hz, J=1.6 Hz, 1H), 6.47 (d, J=16 Hz, 1H), 2.87 (s, 4H).

### Crystal data

**Table:**

C_11_H_9_NO_5_	*F*(000) = 976
*M**_r_* = 235.19	*D*_x_ = 1.499 Mg m^−^^3^
Orthorhombic, *P**b**c**a*	Mo *K*α radiation, λ = 0.71073 Å
Hall symbol: -p 2ac 2ab	Cell parameters from 3779 reflections
*a* = 10.3054 (13) Å	θ = 2.7–27.4°
*b* = 9.2376 (12) Å	µ = 0.12 mm^−^^1^
*c* = 21.892 (3) Å	*T* = 296 K
*V* = 2084.0 (5) Å^3^	Needle, brown
*Z* = 8	0.30 × 0.30 × 0.10 mm

### Data collection

**Table:**

Bruker APEXII CCD area-detector diffractometer	2399 independent reflections
Radiation source: fine-focus sealed tube	1900 reflections with *I* > 2σ(*I*)
graphite	*R*_int_ = 0.041
Detector resolution: 0 pixels mm^-1^	θ_max_ = 27.6°, θ_min_ = 1.9°
φ and ω scans	*h* = −13→13
Absorption correction: multi-scan (*SADABS*; Bruker, 2005)	*k* = −12→12
*T*_min_ = 0.977, *T*_max_ = 0.977	*l* = −28→28
16939 measured reflections	

### Refinement

**Table:**

Refinement on *F*^2^	Primary atom site location: structure-invariant direct methods
Least-squares matrix: full	Secondary atom site location: difference Fourier map
*R*[*F*^2^ > 2σ(*F*^2^)] = 0.035	Hydrogen site location: inferred from neighbouring sites
*wR*(*F*^2^) = 0.082	H-atom parameters constrained
*S* = 1.02	*w* = 1/[σ^2^(*F*_o_^2^) + (0.0285*P*)^2^ + 0.9232*P*] where *P* = (*F*_o_^2^ + 2*F*_c_^2^)/3
2399 reflections	(Δ/σ)_max_ < 0.001
154 parameters	Δρ_max_ = 0.20 e Å^−^^3^
0 restraints	Δρ_min_ = −0.24 e Å^−^^3^

### Special details

**Table:**

Geometry. All s.u.'s (except the s.u. in the dihedral angle between two l.s. planes) are estimated using the full covariance matrix. The cell s.u.'s are taken into account individually in the estimation of s.u.'s in distances, angles and torsion angles; correlations between s.u.'s in cell parameters are only used when they are defined by crystal symmetry. An approximate (isotropic) treatment of cell s.u.'s is used for estimating s.u.'s involving l.s. planes.
Refinement. Refinement of *F*^2^ against ALL reflections. The weighted *R*-factor *wR* and goodness of fit *S* are based on *F*^2^, conventional *R*-factors *R* are based on *F*, with *F* set to zero for negative *F*^2^. The threshold expression of *F*^2^ > σ(*F*^2^) is used only for calculating *R*-factors(gt) *etc*. and is not relevant to the choice of reflections for refinement. *R*-factors based on *F*^2^ are statistically about twice as large as those based on *F*, and *R*- factors based on ALL data will be even larger.

### Fractional atomic coordinates and isotropic or equivalent isotropic displacement parameters (Å^2^)

**Table:**

	*x*	*y*	*z*	*U*_iso_*/*U*_eq_	
O1	0.80658 (9)	0.22692 (10)	0.28201 (4)	0.0261 (2)	
O2	0.89760 (9)	0.55049 (10)	0.10826 (4)	0.0273 (2)	
O3	0.67881 (9)	0.58836 (11)	0.11454 (5)	0.0314 (2)	
O4	0.81461 (10)	0.44760 (11)	−0.00431 (5)	0.0356 (3)	
O5	1.01727 (10)	0.81990 (11)	0.09170 (5)	0.0349 (3)	
C6	0.68207 (13)	0.36659 (14)	0.21024 (6)	0.0237 (3)	
H6A	0.6008	0.3944	0.1960	0.028*	
C7	0.78634 (13)	0.42777 (14)	0.18443 (6)	0.0244 (3)	
H7A	0.8686	0.4048	0.1989	0.029*	
N8	0.89355 (11)	0.63213 (12)	0.05495 (5)	0.0246 (3)	
C9	0.68679 (12)	0.26114 (14)	0.25831 (6)	0.0220 (3)	
C10	0.77299 (13)	0.52965 (14)	0.13407 (6)	0.0233 (3)	
C11	0.96337 (13)	0.76125 (14)	0.04991 (6)	0.0243 (3)	
C12	0.59420 (14)	0.18035 (14)	0.28687 (6)	0.0255 (3)	
H12A	0.5054	0.1829	0.2793	0.031*	
C13	0.85503 (13)	0.56972 (15)	0.00020 (6)	0.0250 (3)	
C14	0.65847 (14)	0.09163 (15)	0.33025 (6)	0.0270 (3)	
H14A	0.6205	0.0249	0.3566	0.032*	
C15	0.78534 (14)	0.12362 (15)	0.32556 (6)	0.0279 (3)	
H15A	0.8503	0.0810	0.3489	0.034*	
C16	0.87575 (15)	0.68378 (15)	−0.04765 (6)	0.0289 (3)	
H16A	0.7934	0.7229	−0.0614	0.035*	
H16B	0.9214	0.6439	−0.0826	0.035*	
C17	0.95729 (14)	0.80146 (15)	−0.01663 (6)	0.0286 (3)	
H17A	1.0438	0.8046	−0.0341	0.034*	
H17B	0.9170	0.8956	−0.0217	0.034*	

### Atomic displacement parameters (Å^2^)

**Table:**

	*U*^11^	*U*^22^	*U*^33^	*U*^12^	*U*^13^	*U*^23^
O1	0.0231 (5)	0.0287 (5)	0.0264 (5)	−0.0003 (4)	−0.0014 (4)	0.0072 (4)
O2	0.0248 (5)	0.0329 (5)	0.0242 (5)	−0.0006 (4)	−0.0008 (4)	0.0097 (4)
O3	0.0272 (5)	0.0337 (5)	0.0334 (5)	0.0032 (4)	−0.0018 (4)	0.0105 (4)
O4	0.0398 (6)	0.0254 (5)	0.0416 (6)	−0.0037 (5)	−0.0043 (5)	−0.0042 (5)
O5	0.0351 (6)	0.0322 (5)	0.0376 (6)	−0.0055 (5)	−0.0022 (5)	−0.0085 (5)
C6	0.0264 (7)	0.0222 (6)	0.0226 (6)	0.0026 (5)	−0.0019 (5)	−0.0008 (5)
C7	0.0253 (7)	0.0243 (7)	0.0235 (7)	0.0016 (6)	−0.0028 (5)	0.0022 (5)
N8	0.0284 (6)	0.0253 (6)	0.0199 (5)	−0.0037 (5)	−0.0009 (5)	0.0052 (5)
C9	0.0222 (6)	0.0232 (6)	0.0208 (6)	0.0025 (5)	−0.0012 (5)	−0.0009 (5)
C10	0.0245 (7)	0.0215 (6)	0.0238 (6)	−0.0014 (6)	−0.0009 (5)	0.0000 (5)
C11	0.0211 (6)	0.0211 (6)	0.0309 (7)	0.0008 (5)	0.0032 (6)	−0.0016 (6)
C12	0.0245 (7)	0.0251 (7)	0.0268 (7)	−0.0017 (6)	0.0018 (5)	−0.0016 (5)
C13	0.0239 (7)	0.0243 (7)	0.0269 (7)	0.0037 (6)	−0.0016 (6)	−0.0025 (5)
C14	0.0363 (8)	0.0227 (6)	0.0220 (6)	−0.0047 (6)	0.0031 (6)	0.0010 (5)
C15	0.0359 (8)	0.0253 (7)	0.0226 (6)	0.0014 (6)	−0.0030 (6)	0.0065 (6)
C16	0.0355 (8)	0.0283 (7)	0.0229 (7)	0.0055 (6)	0.0013 (6)	0.0007 (6)
C17	0.0280 (7)	0.0246 (7)	0.0333 (8)	0.0012 (6)	0.0048 (6)	0.0068 (6)

### Geometric parameters (Å, °)

**Table:**

O1—C15	1.3664 (16)	C9—C12	1.3632 (18)
O1—C9	1.3759 (15)	C11—C17	1.5044 (19)
O2—N8	1.3900 (13)	C12—C14	1.4185 (19)
O2—C10	1.4162 (16)	C12—H12A	0.9300
O3—C10	1.1912 (16)	C13—C16	1.5011 (19)
O4—C13	1.2066 (16)	C14—C15	1.344 (2)
O5—C11	1.1996 (16)	C14—H14A	0.9300
C6—C7	1.3391 (18)	C15—H15A	0.9300
C6—C9	1.4348 (18)	C16—C17	1.533 (2)
C6—H6A	0.9300	C16—H16A	0.9700
C7—C10	1.4561 (18)	C16—H16B	0.9700
C7—H7A	0.9300	C17—H17A	0.9700
N8—C13	1.3880 (17)	C17—H17B	0.9700
N8—C11	1.3974 (17)		
			
C15—O1—C9	106.25 (10)	C14—C12—H12A	126.4
N8—O2—C10	112.43 (9)	O4—C13—N8	123.89 (13)
C7—C6—C9	124.66 (12)	O4—C13—C16	130.41 (12)
C7—C6—H6A	117.7	N8—C13—C16	105.69 (11)
C9—C6—H6A	117.7	C15—C14—C12	106.01 (12)
C6—C7—C10	121.09 (12)	C15—C14—H14A	127.0
C6—C7—H7A	119.5	C12—C14—H14A	127.0
C10—C7—H7A	119.5	C14—C15—O1	111.26 (12)
C13—N8—O2	120.55 (11)	C14—C15—H15A	124.4
C13—N8—C11	115.69 (11)	O1—C15—H15A	124.4
O2—N8—C11	120.93 (11)	C13—C16—C17	105.46 (11)
C12—C9—O1	109.22 (11)	C13—C16—H16A	110.6
C12—C9—C6	133.19 (13)	C17—C16—H16A	110.6
O1—C9—C6	117.58 (11)	C13—C16—H16B	110.6
O3—C10—O2	122.25 (12)	C17—C16—H16B	110.6
O3—C10—C7	130.02 (13)	H16A—C16—H16B	108.8
O2—C10—C7	107.72 (11)	C11—C17—C16	106.07 (11)
O5—C11—N8	124.31 (13)	C11—C17—H17A	110.5
O5—C11—C17	130.25 (13)	C16—C17—H17A	110.5
N8—C11—C17	105.42 (11)	C11—C17—H17B	110.5
C9—C12—C14	107.25 (12)	C16—C17—H17B	110.5
C9—C12—H12A	126.4	H17A—C17—H17B	108.7

### Hydrogen-bond geometry (Å, °)

**Table:**

*D*—H···*A*	*D*—H	H···*A*	*D*···*A*	*D*—H···*A*
C12—H12A···O1^i^	0.93	2.48	3.3533 (17)	156.
C14—H14A···O5^ii^	0.93	2.45	3.3672 (18)	169.

Symmetry codes: (i) *x*−1/2, *y*, −*z*+1/2; (ii) *x*−1/2, *y*−1, −*z*+1/2.
